# A Multiple Length-Scales Nanoimprinting Approach on Nanocrystalline and Strongly Deformed CuZn30 Alloys

**DOI:** 10.1038/s41598-020-58874-y

**Published:** 2020-02-12

**Authors:** Paul Braun, Karsten Durst

**Affiliations:** Physical Metallurgy Division, Materials Science Department, Technische Universität, Darmstadt, Germany

**Keywords:** Nanoscale materials, Techniques and instrumentation

## Abstract

Metallic Nanoimprinting is a new approach to form robust surface structures on metals at various length scales. The shape and size of the formed structures not only depends on the dimensions of the Nanoimprinting die but also the mechanical behaviour of the imprinted material and its microstructure. To characterise the Nanoimprinting process, a multi length-scale-approach was used by varying the cavities (widths between 20 nm and 2.76 µm) as well as the microstructure of the alloy. CuZn30 was used in different cold-worked and heat-treated conditions, with grain sizes from 100 nm up to 277 µm, thus, covering a wide range of hardening behaviours and grain size to cavity width ratios. Experimental results show that the work hardening behaviour as well as the subgrain or grain size have a major influence on the forming characteristics during Nanoimprinting and a nearly ideal plastic behaviour (no work hardening) leads to the largest extrusion heights. For materials with a pronounced work hardening, low extrusion heights were measured for all cavity widths. This work demonstrates the potential of a simple imprinting process to generate surface features on metallic materials with a width <300 nm and an aspect ratio >1.

## Introduction

Surface structures ranging from the millimetre down to the submicron range can be used to functionalise surfaces properties, e.g. improve the frictional properties and wear behaviour of technical surfaces^1^. The main mechanism for decreasing the coefficient of friction and increasing the wear resistance are based on reducing the local contact area^2^ and improving the lubrication condition such as by providing lubrication reservoirs^3^. Extrand *et al*. have shown that the interfacial area between solid and liquid can be reduced up to the point of dewetting by surface structures that consist of pillars and cavities^4^. Tailoring the combination of pillar-geometry, diameter, height and distance can be used to maximize the contact angle. In case of lubricated surfaces, the film lifetime under frictional conditions can be increased by a patterned surface with a sufficient height^5^, that not only provides lubrication reservoirs but also traps for wear particles^3,6^.

For surface structuring in the submicron range, different ablative and forming based techniques ranging from laser ablation to coining are described in literature. Laser based techniques are widely used for structuring surfaces on a variety of materials, typically covering areas from a couple of µm^2^ up to a few thousand mm² ^7^. Depending on the Laser processing technique, such as femtosecond Laser ablation or Laser shock imprinting, different surface structures and functionalities can be realised^8–12^. This includes hierarchical structures for superhydrophobic surfaces or smooth groves that can act as lubrication pockets^4,6^. There is plenty of evidence in literature for the beneficial effect of Laser-patterned surfaces on the frictional properties^13–15^. Some of the works also use a hybrid structuring strategy such as a combination of coined micro-dimples with a Laser fabricated submicron roughness pattern^16^. However, all of these techniques are discontinues processes with only limited upscaling potential that rely on the removal of material from a mostly flat surface area.

In contrast, metal forming processes like coning or imprinting techniques as well as machine hammer peening are based on plastic deformation without material removal^17^. These techniques have the potential to be scaled up to continuous processes that are capable of structuring large surface areas. Bohm *et al*. have produced straight channels and complex rectangular structures in the size between 2 and 20 µm via coining with silicon dies on copper, aluminium, brass and steel. They report a better transferability of the die structures at larger forming pressures, which is in conflict with the risk of a brittle failure of the silicon die^18^. A stamp with an array of 200 nm diamond pillars was used to generate a hole structure on Titanium by Greer *et al*.^19^. Bleicher *et al*. produced surface structures with a height of roughly 20 µm by using machine hammer peening^20^.

These small-scale metal forming processes are strongly influenced by the involved length scales of the forming tools and the microstructure of the material. Specifically, once the tool structure size reaches the same length scale as the grain size, the flow behaviour of the material is strongly limited, leading to a length scale depended flow behaviour^21,22^. Dahl *et al*. found for aluminium the best forming behaviour in a micro-deep drawing process for the smallest examined grain sizes^23^. Furthermore, the ratio of surface grains in the deformed volume to those without surface contact is strongly increasing at small scales, which can eventually increase frictional forces^24^. Against this backdrop, it is apparent that small-scale metal forming processes are strongly influenced by the microstructure of the used substrate and its mechanical properties as well as the dimensions of the tools being used. This is illustrated by the work of Shimizu *et al*.^25^ who investigated the influence of the grain size (Cu with grain sizes between 11 µm and 45 µm) on the transferability in micro-coining process in 200 µm wide cavities. They observed that with a decreasing grain size the surface roughness of the formed structures is reduced. Applying ultrasonic vibrations reduced the surface roughness even further and lead to a strong increase in the forming height of the structures, which was attributed to a reduction in frictional forces. A similar result in combination with a decrease of forming forces was reported by Wang *et al*. in a micro-blanking process with copper foils^26^. In 2007 Wang *et al*. found a minimum in extrusion height for grain sizes in the range of double the cavity width, whereas for a cavity width about 10 times bigger than the grains, the forming behaviour becomes independent of the cavity size^27^. In a later work, a minimum in the filling behaviour was found when the grain size equals the cavity width for grain sizes in the range of 50 µm up to 200 µm. Furthermore, a transition from micro-coining to micro-extrusion was observed for cavities larger than 300 µm^28^.

Nanoimprints with a FIB-milled flat punch tool on polymeric films on a silicon substrate was performed by Cross *et al*. already in 2007^29^. Durst and Hofmann *et al*. have shown, that this technique is also applicable to bulk metals. Furthermore, they were able to show that plastic flow into submicron scale cavities is possible, even though the grain size is much larger than the cavity width^30^. They found that the macroscopic work hardening behaviour is the predominant factor governing the cavity filling behaviour. It was discussed, that an ideal plastic behaviour (no work hardening) leads to a small plastic zone, which favours the flow into the cavities. Ast and Durst later observed a correlation between grain size and surface quality of the formed structures after Nanoimprinting with smaller grain sizes leading to a smoother surface structure^31^. A strong effect of the grain size was also reported by Dhal *et al*. for micro-deep drawing of aluminium. They discussed, that in an ultrafine grained material grain boundary mediated plasticity is predominant and no plastic localisation happens^23^.

**Figure 1:**
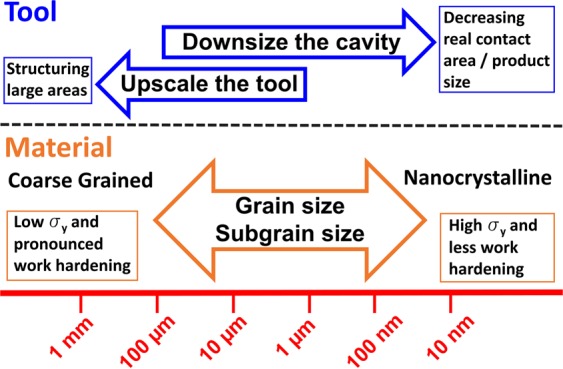
Schematic representation of the different involved length scales for the material selection and variation of the tool geometries.

All previous studies on imprinting have mostly used one die and different coarse grain sizes and only Durst *et al*. and Ast *et al*. used nanocrystalline materials, which are summarized in Table 1. Furthermore the experiments with nanocrystalline materials are limited to electroplated nickel where segregations influence the mechanical behaviour^32,33^. In this new approach, the Nanoimprinting process is analysed for a wide range of microstructural sizes and forming die geometries, employing a multi-length-scale approach (Fig. 1). Thereby several orders of magnitude in the size range between microstructural length scale and forming geometry are covered. Moreover, besides using nanocrystalline materials for the forming process, also cold-worked samples with a dislocation subgrain structure are assessed. Cold working is a common processing for metals and could be easily integrated in a nanoimprinting line for surface structuring. Furthermore, the influence of dynamic and static loading on the nanoimprinting process is analysed as well.

**Table 1:** Cavity and grain sizes of previous studies.

Study	Cavity width	Grain sizes
Wang *et al*.^27^	40–120 µm	16–100 µm
Durst *et al*.^30^	350 nm	20 nm - SX
Ast *et al*.^31^	650 nm	80 nm - SX
Shimizu *et al*.^25^	200 µm	11–45 µm
Wang *et al*.^28^	50–500 µm	13–490 µm

**Figure 2:**
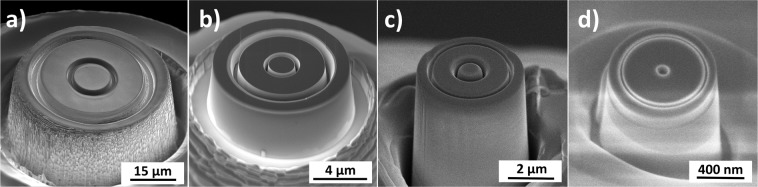
FIB-fabricated rotation-symmetric Diamond Nanoimprinting-dies with different diameters: (**a**) 50 µm, (**b**) 10 µm, (**c**) 5 µm, (**d**) 1 µm.

The experimental approach of this work is to use different FIB-processed diamond tools (diameters between 1 and 50 µm with circular cavities from 20 nm up to 2.76 µm in width) that are pressed into the sample surfaces using a dynamic nanoindentation approach, see also Fig. 2. The geometry of the FIB-processed tools was selected in such a way that a self-similar scaling of the tool and cavity sizes is achieved. The grain size of the used CuZn30 samples was varied between 100 nm (nanocrystalline NC), 3 µm (fine grained FG) and 277 µm (coarse grained CG), by using High Pressure Torsion (HPT)^32^ and subsequent annealing (Fig. 4). With the change in microstructure, also a change in mechanical properties from low yield stress and high work hardening rates in the CG condition to high yield stress and low work hardening rate in the NC condition is simultaneously achieved (Fig. 5). Due to the independent variation of grain size and cavity width a comparison between single- and polycrystalline deformation is possible at different absolute length scale.

**Figure 3:**
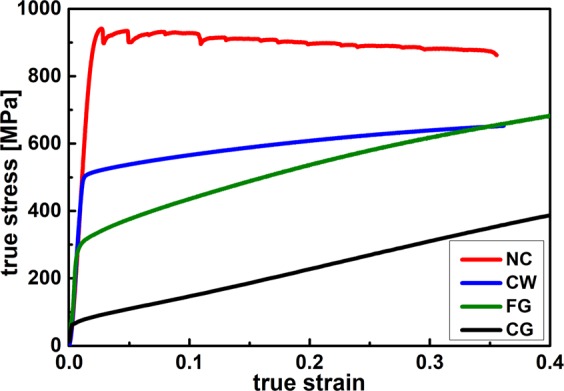
Line scan of an Imprint. h_ind_ marks the plastic indentation depth and h_ext_ the extrusion height – the height the material was extruded into the cavity. Corresponding SEM pictures are found in Fig. 6.

**Figure 4:**
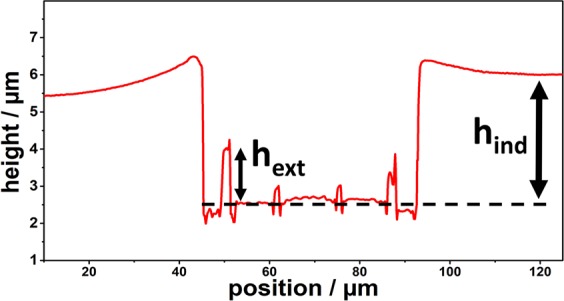
SEM micrographs of the CuZn30 samples. (**a**) Inverse Pole figure map of the nanocrystalline sample measured with transmission Kikuchi diffraction. (**b**) Inverse Pole figure map of the HPT sample after 30 sec of heat treatment at 570 °C measured with EBSD. (**c**) Inverse Pole figure map of the cold-worked sample measured with EBSD. (**d**) Light optical micrograph of the coarse-grained condition etched with Heyn etchant.

**Figure 5:**
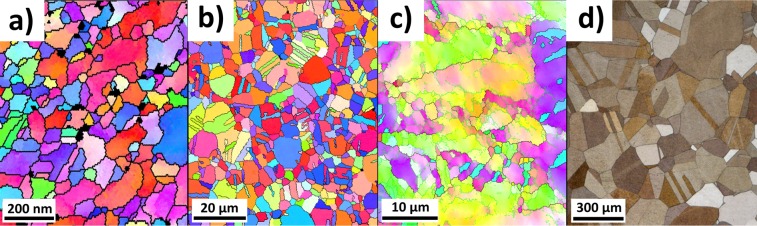
Representative true stress-strain data from the different examined microstructures of CuZn30.

## Experimental Setup

### Materials and sample preparation

The alloy CuZn30 was used as a reference material, with an initial recrystallized coarse-grained microstructure. High Pressure Torsion was used to generate a nanocrystalline microstructure^34^. For this purpose, discs with a diameter of 20 mm and an initial height of 2 mm (approx. 1.5 mm after HPT) were deformed to 12 revolutions with a force of 1400 kN. After HPT processing, the materials exhibits a small grain size and a high dislocation density at the grain boundaries^32,35^. Heat treatments were performed to 1) increase the grain size 2) decrease the dislocation density within the alloy. For this purpose heat treatment at T = 570 °C for t = 30 s are performed. To generate a coarse-grained microstructure with a high dislocation density, cylindrical samples of the as received material were deformed in a compression test up to an engineering strain of 60%. For microstructural characterisation a scanning electron microscope (MIRA 3, TESCAN) equipped with an EBSD system (DigiView IV, EDAX) was used.

### Mechanical properties

Compression tests were performed for at least 5 samples, with a diameter of 2 mm and a height of 3 mm in a universal testing machine Instron 5967 equipped with a 30 kN load cell using a constant displacement rate corresponding to an initial strain rate of ${10}^{-3}\,\frac{1}{s}$. A Teflon tape was placed between the sample and the anvils to reduce the friction.

### Tools

Flat punch Nanoimprinting tools with diameters between 1 µm and 50 µm were fabricated from raw diamond tips (SYNTON-MPD) by focused ion beam milling in the Application Lab from TESCAN Bruno, Czech, using Ga and Xe based FIBs. The tools are shown in Fig. 2.

An aspect ratio (height/diameter) below ½ was used for all forming tools in order to prevent fracture during the imprinting process. Afterwards, two ring cavities with different diameters were milled into the surface of the flat punch. The depths of the cavities were chosen to be double the width. The different tool geometries are summarized in Table 2, here ETX means: Extrusion Tool and the number X indicates the diameter in µm. Due to the beam shape and redeposition during FIB-milling, the walls of the cavities are not perfectly straight. Especially for the 50 µm tool, a high surface roughness is found and the highest frictional forces are expected for this tool.

**Table 2:** Diameter of the Nanoimprinting tools.

Tool		Tool diameter [µm]	diameter outer ring [µm]	cavity width [nm]
ET50	Fig. 2(a)	47.5	36,4	2760
ET10	Fig. 2(b)	10.4	7.2	688
ET5	Fig. 2(c)	4.69	3.6	290
ET1	Fig. 2(d)	1.08	0.75	20

### Imprinting and evaluation

The imprinting experiments were performed using a Nanoindenter (Keysight G200) equipped with a high-load cell (up to 10 N) in continuous stiffness method (CSM) and load controlled (LC) mode. The CSM method was used for all tools with an oscillation frequency of 40 Hz and a harmonic displacement amplitude of 2 nm^36^, whereas LC experiments were only performed with the 10 µm tool for a comparison of both methods. A goniometer stage was used to ensure good alignment between the flat punch Nanoimprinting tool and the specimen surface.

**Figure 6:**
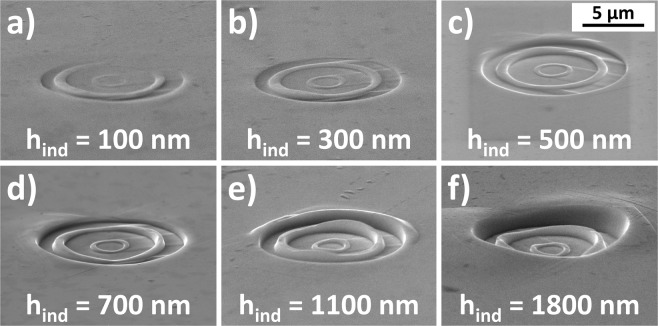
Series of deformation with different indentation depth [(**a**) 100 nm; (**b**) 300 nm; (**c**) 500 nm; (**d**) 700 nm; (**e**) 1100 nm and (**f**) 1800 nm] in the cold worked sample with the 10 µm tool.

The plastic depth and the cavity extrusion height was analysed with a Laser Scanning Microscope LEXT 4100 from Olympus. The extrusion height ${h}_{ext}$ and plastic depths ${h}_{ind}$ were measured according to Fig. 3 (corresponding SEM images are found in Fig. 6). All symbols used in this work are summarized in Table A1 in the appendix. For analysing the extrusion behaviour, the extrusion height of the outer ring was studied as a function of the plastic indentation depth.

## Results

### Characterisation of the used material

Figure 4 shows typically SEM EBSD and light optical micrographs of the CuZn30 samples after different work hardening and heat treatments. For the coarse grained sample a grain size of 277 µm was measured (Fig. 4d). After HPT-processing a grain size of 100 nm was determined with Transmission Kikuchi Diffraction (TKD)^34^. The heat treated HPT sample (fine grained) exhibits a grain size of 2.96 µm. For the cold worked sample a dislocation cell size of 390 nm was determined, while the grain size based on high angle boundary is similar to the coarse grained state (≈280 µm). The grain sizes were quantified using either the line intercept method or the equivalent circle diameter based on the TKD/EBSD maps and are summarized in Table 3.

**Table 3:** Grain size and mechanical properties of the used CuZn30 samples.

Sample	Grain size	Dislocation subgrain size	Hardness [GPa]	Flow Stress [MPa]	Hardening exponent
CG (coarse grained)	277 ± 20 µm		1.11 ± 0.04	63 ± 5	0.71
CW (Cold worked = 60% plastic strain)	277 ± 20 µm	390 ± 25 nm	2.04 ± 0.14	500 ± 10	0.09
NC (HPT processed)	100 ± 50 nm		3.18 ± 0,02	940 ± 12	−0.02
FG (HPT + 30 sec @570 °C)	2.96 ± 1.7 µm		1.37 ± 0.03	310 ± 12	0.29

Representative compression true stress-strain curves of the different microstructural conditions are shown in Fig. 5. The Hollomon strain hardening coefficient $n$ is calculated based on the stress-strain data using equation (1)^37^.

1 $$n=\frac{\partial \,log({\sigma }_{true})}{\partial \,\log ({\varepsilon }_{true})}=\frac{{\varepsilon }_{true}}{{\sigma }_{true}}\frac{\partial {\sigma }_{true}}{\partial {\varepsilon }_{true}}$$

The coarse-grained (CG) state exhibits a strong work hardening with a strain hardening exponent of 0.73, which is reduced to 0.09 in the cold worked (CW) specimen after 60% prestrain. The nanocrystalline (NC) specimen subjected to HPT processing shows a nearly ideal plastic behaviour with a slight strain softening resulting in an exponent of −0.02. The fine grained (FG) samples subjected to HPT and annealing show an intermediate hardening exponent of 0.29. The grain sizes and the mechanical properties are summarised in Table 3.

### Extrusion height vs Indentation depth

**Figure 7:**
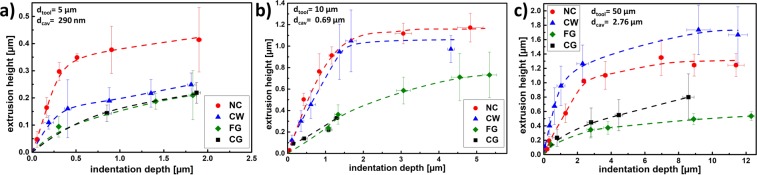
Extrusion height as a function of the residual indentation depth for three different tools. (**a**) 5 µm tool; (**b**) 10 µm tool; (**c**) 50 µm tool.

Figure 6 shows a series of SEM images on the evolution of the imprinted geometry at different depths for the cold worked (CW) sample, using a die with a diameter of 10 µm. The corresponding quantitative extrusion data is plotted in Fig. 7b.

During the initial contact (indentation depth ~ 100 nm), the structure is only partly imprinted, due to slight misalignment between tool and surface. The slight misfit is compensated by plastic flow (here for a h_ind_ = 300 nm) and the extrusion height increases linearly with the indentation depth (Fig. 7b). The linear regime, where the extrusion height increases proportional to the indentation depth ends for this experiment at an indentation depth of 1100 nm. At larger indentation depths, the extrusion does not increase strongly, reaching a saturation regime and the formed structure is pushed deeper into the surface (h_ind_ = 1800 nm). The same behaviour, i.e. initial alignment followed by linear extrusion and approaching a saturation regime, is found for all tools and material combinations.

In Fig. 7 the extrusion data is plotted as a function of the indentation depth for all materials and the 5 µm, 10 µm and 50 µm tools. All data follow the same trend, but at different absolute depth and extrusion height values.

Moreover, the saturation regime is most pronounced for the materials showing the largest extrusion height. In general, the highest extrusion heights are achieved for the nanocrystalline sample with the exception of the 50 µm tool. The cold worked sample shows a good cavity filling for the 10 and 50 µm tool and even exceeds the nanocrystalline sample with the 50 µm tool. The fine grained sample and the coarse-grained sample show a similar extrusion behaviour: Both exhibit a low increase in extrusion height, whereas a saturation is not clearly observable.

## Discussion

### Relative extrusion height – linear regime

**Figure 8:**
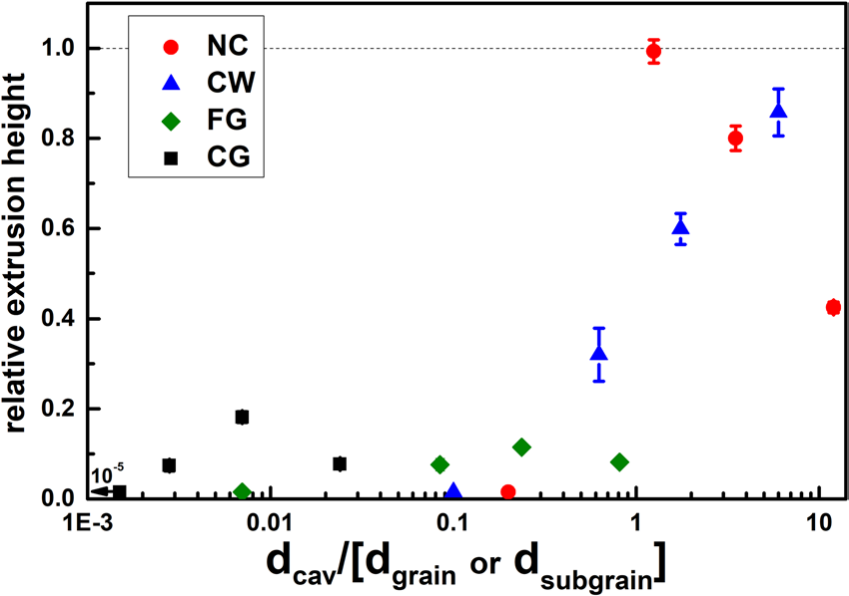
Relative extrusion height versus the cavity width normalised with respect to grain or dislocation cell size. For each microstructural state, the data from the small tool are found on the left hand side, followed by the larger tools, e.g. 1 µm, 5 µm, 10 µm and 50 µm tool.

To compare the flow behaviour into the cavities of the different microstructural states, the relative extrusion height ${{\boldsymbol{h}}}_{{\boldsymbol{rel}}}$ (calculated by equation (2)) is analysed as the linear slope of the extrusion height versus the indentation depth (in the linear regime). To compare the different die geometries, the relative extrusion height is plotted as a function of the cavity width d_cav_ normalised with respect to the grain size d_grain_ or dislocation subgrain size d_subgrain_ in Fig. 8. The ratio of cavity size to grain size can be understood as the number of grains, that are required to fill the width of the cavity, which varies from <10^−5^ up to 12 for the investigated microstructural conditions and geometries. For each microstructural state, the data corresponding to the smallest tool are found on the left, e.g. 1 µm, 5 µm, 10 µm and 50 µm tool from left to right.

2 $${h}_{rel}=\frac{\partial {h}_{ext}}{\partial {h}_{ind}}$$

**Figure 9:**
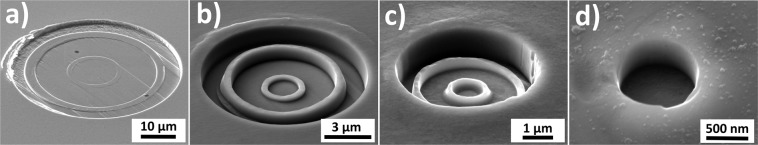
Extrusion geometries with maximum aspect ratio for different tools (**a**) 50 µm; (**b**) 10 µm; (**c**) 5 µm; (**d**) 1 µm tool (no extrusion); near to the sweet spot of extrusion for the NC sample.

It is obvious, that the cavity filling behaviour decreases as soon as the cavity width becomes smaller than the grain size. This also holds for the nanocrystalline sample where no extrusion could be generated with the 1 µm tool where the ratio of cavity width to grain size is about 0.2 (Fig. 9(d)). This tool has a cavity width of 20 nm and no plastic flow was obtained for any sample into the cavities. However, it is possible to produce holes with a diameter of 1 µm with that stamp.

It is noteworthy, that both the CG and the CW-state have the same grain size, however, the CW condition exhibits extrusion heights comparable to the NC state. This indicates that the subgrain size needs to be considered as the relevant microstructural length scale in the Nanoimprinting process. Both the fine-grained state (FG) deformed with the 50 µm tool and the cold-worked state (CW) deformed with the 5 µm tool, exhibit a similar ratio of cavity width to grain size of 0.7–0.8. However, the CW-state reaches a relative extrusion height of 0.32, whereas the FG state shows a much lower value of only 0.05. Consequently, not only the grain or subgrain size is important, but also the local flow behaviour of the material that is strongly influenced by the dislocation density and dislocation mobility. Furthermore, the macroscopic work hardening behaviour needs to be considered, too. The higher work hardening rate of the FG state (n = 0.29), leads to large stress gradients in the plastic zone and the deformation is shifted to the far field resulting in a sink-in, see also Fig. 6 (h_ind_ = 1800 nm). This limits the flow into the cavities, resulting in small extrusion heights. A low work hardening exponent (n = 0.09 for CW) leads to small stress gradients below the indenter and facilitates a material flow commences into the cavities.

The specimen with the lowest strain hardening exponent is the nanocrystalline state produced via HPT. It not only contains a very high density of grain boundaries, which have been generated by the severe plastic deformation process, but also a high density of mobile dislocation are to be expected at the grain boundaries. The nanocrystalline sample reaches thereby a maximum observed relative extrusion height of nearly 1 with the 5 µm tool. Another comparison can be drawn between the NC-state using the 5 µm tool and the CW state using the 10 µm tool, which represent similar cavity to (sub)grain size ratios of 1.25 and 1.75, respectively. Yet, the cold-worked specimen reaches a much smaller relative extrusion height as compared to the NC state. This effect can again be attributed to the difference in the strain hardening behaviour of these conditions.

A decreasing of the forming capability by using bigger cavities that is observable in Fig. 8 for the nanocrystalline sample was also reported by Wang *et al*.^27^. At a certain cavity size, which depends on the grain size, a macroscopic mode of deformation is reached, where the forming behaviour becomes self-similar and scale independent. Hence, a constant relative extrusion height, regardless of the tool dimensions is expected. For the NC material this state could be achieved for the 50 µm tool, while for the cold worked condition even larger cavities width seem to be required. However, frictional effects can also cause a decrease of the relative extrusion height (when the extruded material is in contact with the cavity wall), especially when considering the differences in surface roughness of the tools (see subchapter 4.3).

### Maximum extrusion heights

The NC and CW material exhibit the largest relative extrusion height, which makes these conditions promising candidates from an application point of view. The end of the linear extrusion regime marks a sweet spot in the nanoimprinting process for these material conditions. At this position, the highest surface structures with a low sink-in can be achieved. The FG and CG state exhibit only a limited extrusion height and the structures starts to sink-in already at the initial contact. The aspect ratio (extrusion height divided by the cavity width) at this point can be considered as a benchmark for the imprinting behavior of certain material conditions and cavity widths and is shown in Table 4. Exemplarily SEM-micrographs of these conditions are given in Fig. 9 for the NC sample.

**Table 4:** Optimized extrusion heights for the different tools on the nanocrystalline and cold-worked sample.

Tool	nanocrystalline		Cold-worked	
	Indentation depth [µm]	Aspect ratio	Indentation depth [µm]	Aspect ratio
ET5	0.51 ± 0.02	1.2 ± 0.05	0.41 ± 0.04	0.55 ± 0.1
ET10	1.17 ± 0.08	1.3 ± 0.09	1.37 ± 0.08	1.37 ± 0.1
ET50	2.38 ± 0.03	0.37 ± 0.01	2.3 ± 0.35	0.46 ± 0.1

The data, especially the SEM-pictures (Fig. 9), show that Nanoimprinting can generate surface structures in the submicron range with an aspect ratio higher than 1. The cold-worked sample shows an aspect ratio similar to the nanocrystalline sample at the sweet spots for tools with a diameter larger than 10 µm. However, an aspect ratio of 1 for a cavity width of 290 nm can only be achieved with the nanocrystalline sample (Fig. 9c). Interestingly, the largest tool achieves the worst aspect ratio, which in turn makes Nanoimprinting particularly interesting for structures in the submicron regime. Yet, there is a lower size limit for achievable structures, which is demonstrated by the 1 µm tool with a cavity width of 20 nm where no extrusion could be generated with any of the investigated material conditions (Fig. 9d).

### Static versus dynamic imprinting

**Figure 10:**
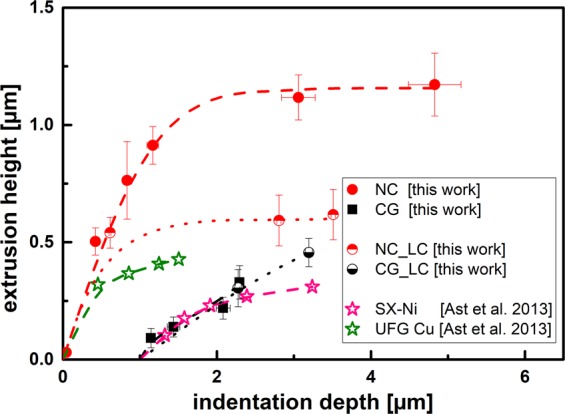
Comparison of extrusion data between Ast *et al*.^31^ and the current work using the 10 µm tool. The CG and SX-Ni data is offset by 1 µm for the indentation depth.

A similar approach to this work for studying the extrusion behaviour was followed by Ast *et al*. using a punch with a diameter of 8.2 µm and a cavity width of 650 nm^31^. Those numbers are very similar to the 10 µm tool used in this work with a cavity width of 688 nm. Comparing the results in Fig. 10, it is striking that the initial slope of the UFG materials studied by Ast *et al*. is very similar to the nanocrystalline sample studied in this work. Furthermore, it is apparent that the saturation extrusion heights achieved in this study are significantly larger. One possible reason for the discrepancy in extrusion heights could be the dynamic method (CSM) that was used in this work, which potentially reduces the friction between the cavity walls and the extruded material. The frictional effect is most pronounced, once the extruded material is in contact with the cavity walls, which does occur at the large extrusion heights that are achieved during imprinting of the NC or UFG materials. This effect becomes apparent when comparing the deviation from the linear extrusion regime for the NC sample under dynamic (NC) and static loading (NC_LC) condition. Both methods yield the same slope of the initial extrusion height, yet, under static loading, an early deviation from the linear regime (similar to the UFG sample by Ast *et al*.) and a much smaller saturation height is observed. A similar trend would also be expected for the coarse grained samples (SX-NI, CG_LC, CG), yet, static (CG_LC) and dynamic (CG) extrusion data are almost identical. This can be attributed to the comparably small extrusion heights of these conditions leading to a very limited contact area between extruded material and cavity walls, thus, reducing the impact of frictional effect during the imprinting process.

### Mechanistic view on the extrusion formation

Summing up all the findings it can be concluded that the extrusion mechanisms during nanoimprinting are a complex interaction of different flow mechanism and regimes. As can be seen in Fig. 6, first the indenter establishes contact with the surface and then the material is extruded into the cavities, until a saturation in extrusion height is achieved and the deformed volume sinks into the surface. The sink-in behaviour is thereby one of the limiting factors in the extrusion process, and is mainly governed by the work hardening of the material. Coarse grained materials with a low yield strength and strong work hardening show a sink-in, starting from the initial contact and thereby forming only low extrusion heights (Fig. 7). The formation of the extrusion on the other hand depends on the cavity width to grain size ratio. For a grain size larger than the cavity width, only little plastic flow into the cavity occurs, while for a cavity width to grain size ratio >1 (i.e. multiple grains fit into the cavity), plastic flow occurs easily, leading to the formation of a large extrusion height (Fig. 8). The relevant length scale in that context is either the high angle grain boundary spacing (like in the case of the NC material) or the spacing of the subgrains after coldworking. The maximum extrusion height is limited by the work hardening behaviour and the friction between the cavity wall and the material. The latter can be reduced by dynamic imprinting with a superimposed oscillation that has proven to generate larger maximum extrusion heights than under quasi-static loading conditions (Fig. 10).

**Figure 11:**
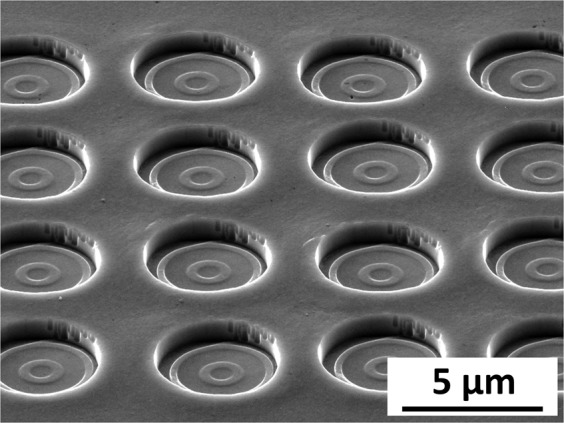
Array of imprints done with the 5 µm tool as an example for creating functionalized technical surfaces by metallic Nanoimprinting. The spacing from centre to centre of the imprints is approx. 7 µm.

This work has shown, that it is possible to use Nanoimprinting in various length scales to structure metallic surfaces. While the tools and the nanoindentation based setup of this work are only able to structure very small areas, different approaches such as machine hammering would be able to create an array of imprints to generate a functionalized surface as exemplarily shown in Fig. 11. Another option to structure technical surfaces is to upscale the tool, i.e. including a large number of single pillars, as shown by Greer *et al*.^19^, with cavity structures on the pillars. Such larger tools could be used to structure a bigger surface area in a single stroke.

## Conclusions

The Metallic Nanoimprinting process was studied using a multi length-scale-approach in terms of different tool dimension with ring cavities and different grain sizes. CuZn30 was used as substrate in different cold-worked and heat-treated conditions to change the hardening behaviour and the cavity width to grain size ratio. Of particular interest was the relative extrusion height given as the slope in the linear regime of the extrusion data and the saturation extrusion height. From the results of the experiments, the following conclusions can be drawn:

- The work hardening behaviour has the strongest effect on the forming characteristics during Nanoimprinting. Materials exhibiting strong work hardening show low extrusion heights for all examined cavity widths, with little difference between the quasi-single crystalline (CG) and polycrystalline material (FG). This can be attributed to the sink-in behaviour that occurs during the imprinting process.
- Materials with little to no work hardening, lead to a confinement of the plastic deformation in close vicinity of the contact, which improves the flow of material into the cavities. Here, the cavity width to grain size ratio controls the extrusion height. For grain size to cavity width ratios <1, there is little to no plastic flow into the cavities, whereas at ratios >1, plastic flow occurs resulting large extrusion height.
- Cold worked material with a dislocation cell structure exhibits large extrusion heights due to the decreased work hardening capability of the material, combined with a small subgrain size. However, for subgrain sizes above the cavity width, low extrusion heights are formed and nanocrystalline materials are required to create even smaller surface structures.
- The saturation extrusion height is strongly limited by the friction between the cavity wall and the extruded material. Dynamic nanoimprinting leads to a reduction in friction and therefore to larger saturation extrusion heights.
- The smallest extruded ring geometries that could be realized have a wall thickness of 290 nm with a height of roughly 300 nm using a nanocrystalline sample processed by high pressure torsion as substrate material.

## Appendix

**Table A1:** Symbols used in this work.

Symbol	Meaning
h_ind_	indentation depth
h_ext_	extrusion height
h_rel_	relative extrusion height
d_tool_	tool diameter
d_cav_	cavity width
d_grain_	grain size
d_subgrain_	dislocation subgrain size
${\sigma }_{true}$	true stress
${\varepsilon }_{true}$	true strain
$n$	Hollomon-exponent
